# Renal pseudoaneurysm after calculous pyelonephritis

**DOI:** 10.1002/iju5.12711

**Published:** 2024-02-27

**Authors:** Yuri Koyama, Tetsuya Yumioka, Hirofumi Ohno

**Affiliations:** ^1^ Division of Urology, Department of Surgery, Faculty of Medicine Tottori University Yonago Tottori Japan; ^2^ Department of Urology Matsue Red Cross Hospital Matsue Shimane Japan

**Keywords:** angioembolization, infection, pyelonephritis, renal pseudoaneurysm, ureteral calculi

## Abstract

**Introduction:**

Renal pseudoaneurysms reportedly occur after partial nephrectomy, renal trauma, or percutaneous procedures. Renal pseudoaneurysms can also occur after renal inflammation; however, such cases are rare and seldom reported.

**Case presentation:**

A 53‐year‐old man presented to our emergency room with a 3‐day history of fever and right back pain. A blood sample revealed severe inflammation and computed tomography showed an 8 mm diameter stone in the right middle ureter and hydronephrosis. The patient was diagnosed with calculous pyelonephritis and underwent emergency ureteral stenting and antibiotic therapy. On day 8 of hospitalization, hematuria and right back pain developed, and on day 9 bladder tamponade and anemia developed. Contrast‐enhanced computed tomography revealed a ruptured pseudoaneurysm, and the patient underwent successful angioembolization. The patient was discharged on day 23.

**Conclusion:**

We report a case of a renal pseudoaneurysm possibly caused by calculous pyelonephritis.

**Abbreviations & Acronyms**
CT: computed tomography
f‐TUL: flexible transurethral lithotripsy

**Keynote message**

Hematuria after pyelonephritis, although rare, should be considered as a possible indication of a pseudoaneurysm.

## Introduction

Renal pseudoaneurysms reportedly occur after partial nephrectomy, renal trauma, or percutaneous procedures. Renal pseudoaneurysms can also occur after renal inflammation; however, such cases are rare and seldom reported. Herein, we report a case of renal pseudoaneurysm formation, possibly due to acute pyelonephritis, associated with urinary obstruction caused by ureteral calculi.

## Case presentation

A 53‐year‐old man with a 3‐day history of fever and right back pain was admitted to our hospital. Ten years before admission to our hospital, he was diagnosed with urinary stones but self‐interrupted. Physical examination indicated a high fever of 39°C and right costovertebral angle tenderness. He had no history of hypertension; however, his blood pressure was 140/105 mm Hg despite having sepsis. The patient's blood sample showed inflammation (WBC 21 600/μL, CRP 43.19 mg/dL, procalcitonin 140 ng/mL), decreased renal function (BUN 54.7 mg/dL, Cre 2.74 mg/dL), and abnormal coagulation (PT‐INR 1.20, APTT 41.7 s). Pyuria was observed.

**Fig. 1:**
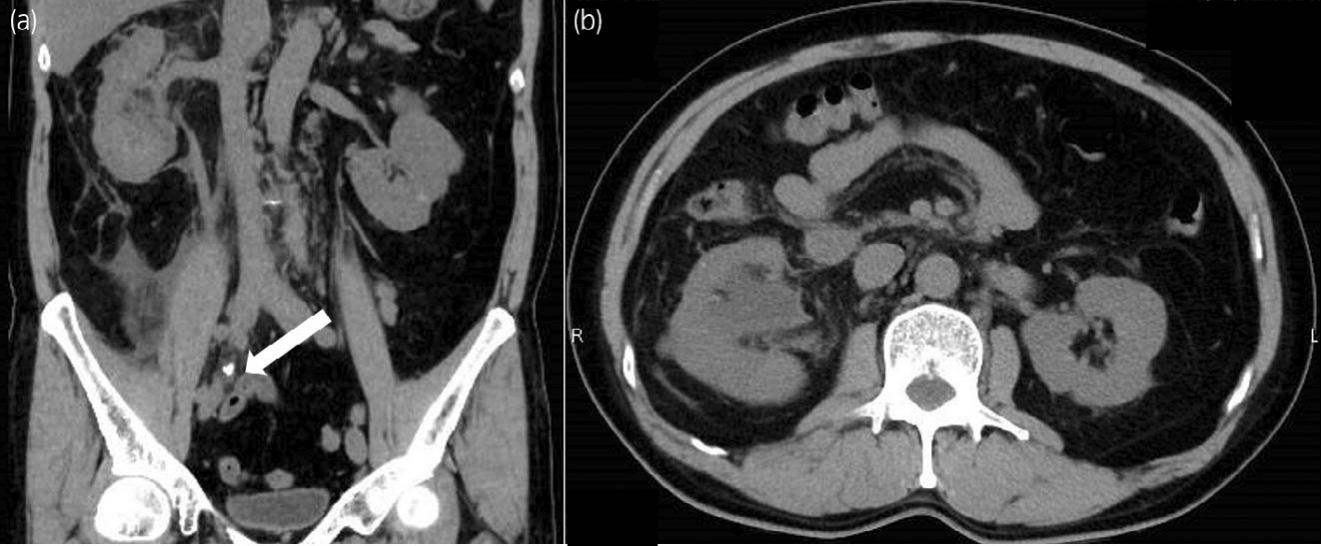
Non‐contrast CT at initial examination demonstrates an 8 mm diameter stone in the right middle ureter and hydronephrosis. (a) Coronal; (b) Axial.

Computed tomography (CT) revealed an 8 mm diameter stone in the right middle ureter and hydronephrosis (Fig. 1).

**Fig. 2:**
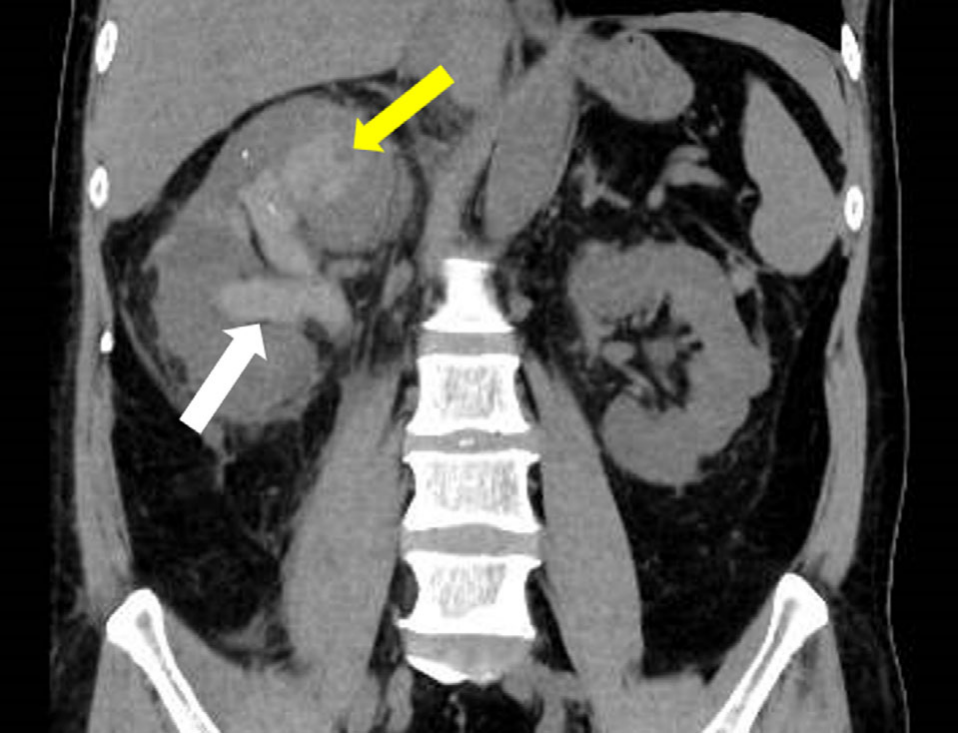
Non‐contrast CT on day 8 demonstrates a hematoma extending from the right renal pelvis to the ureter (white arrow). A high‐density area was observed within the renal parenchyma near the superior calyx (yellow arrow). There was a clear change when compared to the simple CT at admission.

**Fig. 3:**
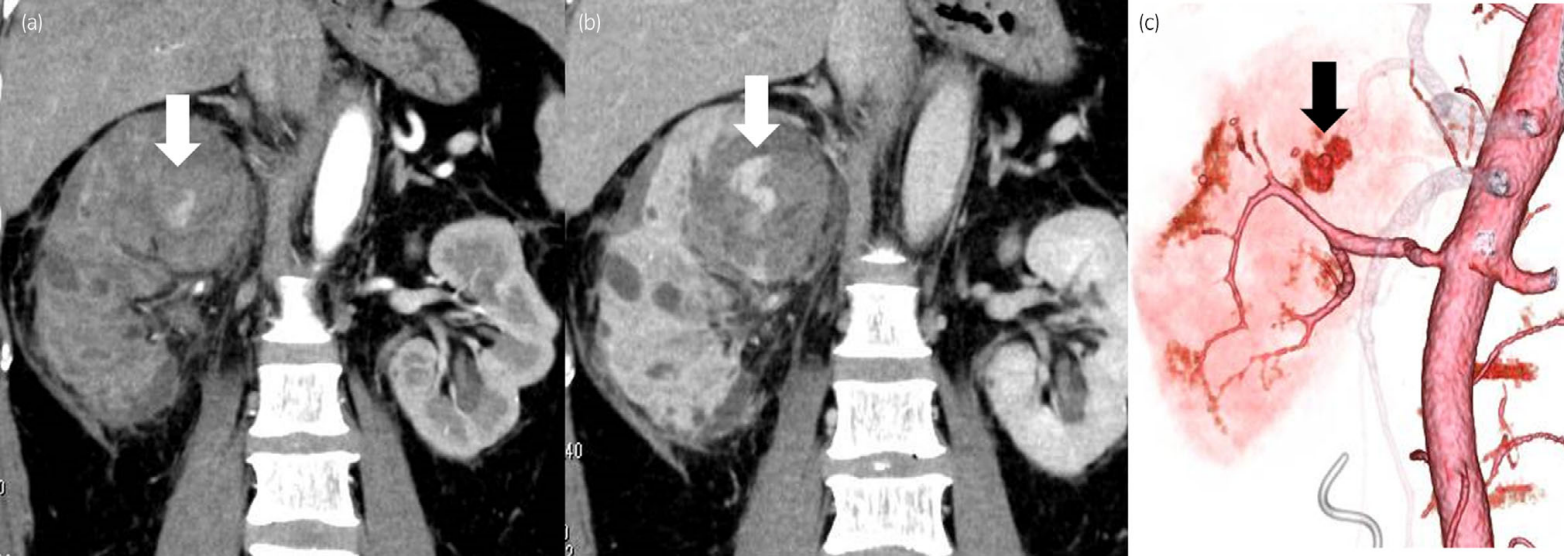
Contrast‐enhanced Computed Tomography on day 9 demonstrates a ruptured pseudoaneurysm. (a) Arterial phase; (b) Venous phase; (c) 3D‐Compued Tomography.

The patient was diagnosed with acute pyelonephritis associated with urinary obstruction due to ureteral calculi, and we performed emergency ureteral stenting, and broad‐spectrum antibiotic therapy was initiated. On day 5, *Staphylococcus aureus* (*MSSA*) was detected in blood and urine cultures, and the antibiotic was changed to cefazolin. Hematuria did not occur after stenting but appeared on day 8. Due to persistent fever, non‐contrast CT was performed on day 8. This revealed a hematoma extending from the right renal pelvis to the ureter (Fig. 2). We replaced the right‐side single‐J catheter and changed to a broad‐spectrum antibiotic. On day 9, the patient had right back pain and bladder tamponade (Hemoglobin −1.9 g/mL). Contrast‐enhanced CT revealed a ruptured pseudoaneurysm and embolization was performed (Fig. 3).

Postembolization angiography revealed no pseudoaneurysms or contrast leakage. The inflammation resolved, and there was no recurrence of bleeding. The patient was discharged on day 23. The patient underwent flexible transurethral lithotripsy (f‐TUL) after hospital discharge.

## Discussion

Renal pseudoaneurysms are a rare condition and they occur following arterial injury in the kidneys.^1^ Most reported cases were due to renal trauma of an iatrogenic origin. Recently, several reports have described aneurysms after partial nephrectomy.^2^ However, there are few reports of renal pseudoaneurysms caused by infections. To our knowledge, the only reports of pseudoaneurysms suspected to be caused by infection are those overlying renal trauma,^3^ ruptured pseudoaneurysms after TUL for calculous pyelonephritis,^4^, ^5^, ^6^ and renal abscess.^7^

Infected aneurysms were first reported by Willam Osler in 1885 and are said to rupture more frequently than non‐infectious aneurysms.^8^ Although the detailed mechanism of aneurysm formation remains unclear, bacteria tend to adhere to injured arterial intima, leading to infectious vasculitis, which precipitates arterial wall collapse and subsequent pseudoaneurysm formation.^9^ The routes of infection of the arterial wall include, an origin of infective endocarditis, adjacent foci of infection, bacterial infection of damaged intima from the bloodstream, and trauma.^10^

We considered the possibility that it could have formed because of renal injury associated with stent placement. However, as there was no hematuria after stenting, this was ruled out.

Another possibility is that an original renal pseudoaneurysm may have ruptured because of ureteral stenting or calculous pyelonephritis. As the CT scan on admission was non‐contrast, we could only confirm the absence of an obvious aneurysm. There was no history to suggest a cause, such as renal trauma or extracorporeal shock wave lithotripsy. There were clear changes compared to the simple CT scan at admission and on day 8.

There was a report of a renal artery pseudoaneurysm that was formed and ruptured 1–2 weeks after infection of a renal abscess.^7^ As this report is very similar to the present case, we believe that the pseudoaneurysm might have ruptured approximately 2 weeks after infection.

We concluded that the formation and rupture of the pseudoaneurysm were most strongly associated with calculous pyelonephritis (infection). In addition, the presence of hypertension and abnormal coagulation due to sepsis may have contributed to the pseudoaneurysm formation.

Reports on pseudoaneurysms after TUL suggest that increased intrarenal pressure in the renal pelvis could be an underlying factor.^11^ However, if this is the only cause, it should also occur with impacted ureteral stones. Approximately 50% of infectious aneurysms are caused by gram‐positive cocci.^12^ Usually, pyelonephritis is caused by Gram‐negative bacilli; however, in this case, the infection was MSSA, which may have been one of the factors that caused the infected aneurysm. We speculate that an increase in intrarenal pressure in the renal pelvis and the presence of inflammation, which is of sufficient severity to destroy the arterial wall, may increase the risk of pseudoaneurysm formation.

Infected aneurysms are rarely reported in cases of pyelonephritis; however, there are scattered reports of infections in other organs. Pseudoaneurysms of the splenic artery following pancreatitis are well known and are reported to be associated with approximately 10% of chronic pancreatitis cases, of which 2%–10% rupture.^13^ There have also been reports of the rupture of pseudoaneurysms of the pancreaticoduodenal artery after acute pancreatitis, and pseudoaneurysms of the pulmonary artery after pyogenic tonsillitis or lung abscess.^14^, ^15^, ^16^

## Conclusion

We encountered a case of suspected pseudoaneurysm secondary to calculous pyelonephritis. Hematuria after pyelonephritis, although rare, should be considered as a possible indication of a pseudoaneurysm.

## Author contributions

Yuri Koyama: Conceptualization; data curation; formal analysis; investigation; methodology; project administration; validation; visualization; writing – original draft; writing – review and editing. Tetsuya Yumioka: Supervision; writing – review and editing. Hirofumi Ohno: Supervision.

## Conflict of interest

The authors declare no conflicts of interest.

## Approval of the research protocol by an Institutional Reviewer Board

Not applicable.

## Informed consent

Not applicable.

## Registry and the Registration No. of the study/trial

Not applicable.
